# Knockdown of RNA N6-methyladenosine methyltransferase METTL3 represses Warburg effect in colorectal cancer via regulating HIF-1α

**DOI:** 10.1038/s41392-021-00473-y

**Published:** 2021-02-27

**Authors:** Zhou Yang, Yingjun Quan, Yusheng Chen, Yijun Huang, Renhong Huang, Weiping Yu, Dejun Wu, Min Ye, Zhijun Min, Bo Yu

**Affiliations:** 1 Department of General Surgery, Shanghai Key Laboratory of Vascular Lesions Regulation and Remodeling, Shanghai Pudong Hospital, Fudan University Pudong Medical Center, Shanghai, China; 2 Department of General Surgery, Tongren Hospital, Shanghai Jiao Tong University School of Medicine, Shanghai, China; 3 Department of General Surgery, Huashan Hospital Affiliated to Fudan University, Shanghai, China

**Keywords:** Cancer metabolism, Gastrointestinal cancer

**Dear Editor**,

Colorectal cancer (CRC) is an important digestive tract tumor with high malignancy. Nearly one million people suffer from CRC every year with a mortality rate of 33%. Therefore, it is urgent to determine the mechanism for the development of CRC, in order to find more effective diagnosis markers and therapeutic targets.

Various RNA modifications have been identified in both mRNA and non-coding RNAs, with RNA methylation being the most common mRNA modification in eukaryotes. Methylation modifications of RNA are m6A (N6-methyladenosine, 6-methyl adenine) and uridine modification (U-tail), in which m6A is the most common^1^. However, most research in CRC, as well as other tumors, focused on the progression, but rare attention was paid to tumor metabolism. Even when the oxygen supply is sufficient, the tumor cells obtain energy mainly by glycolysis, which is also called aerobic glycolysis or Warburg effect. This abnormal glycolysis of tumor cells promotes rapid glucose uptake, ATP, and lactate production, which is conducive to tumorigenesis. Our study aimed to investigate the role of m6A modification writer METTL3 in Warburg effect and its association with the metabolism of CRC. Therefore, our research focused on the association between m6A modification and Warburg effect in CRC, which is a crucial mechanism for tumor development.

We primarily detected the expression of METTL3 in the TCGA database and clinical CRC specimens. METTL3 accompanied with total m6A levels were significantly increased in CRC specimens compared with paired non-tumor tissues (Fig. 1a, b). Furthermore, we observed the level of METTL3 showed a satisfying prediction factor for both lymph node and distant metastasis, as well as the pathological stages (Fig. 1c–f). We also determined overexpression of METTL3 was associated with poor OS via COX model analysis and Kaplan–Meier analysis (Fig. 1g, Supplementary Table S1). In addition, an obvious co-expression between METTL3 and HIF-1α, the main regulator of Warburg effect, was identified (Fig. 1h, i).

**Fig. 1:**
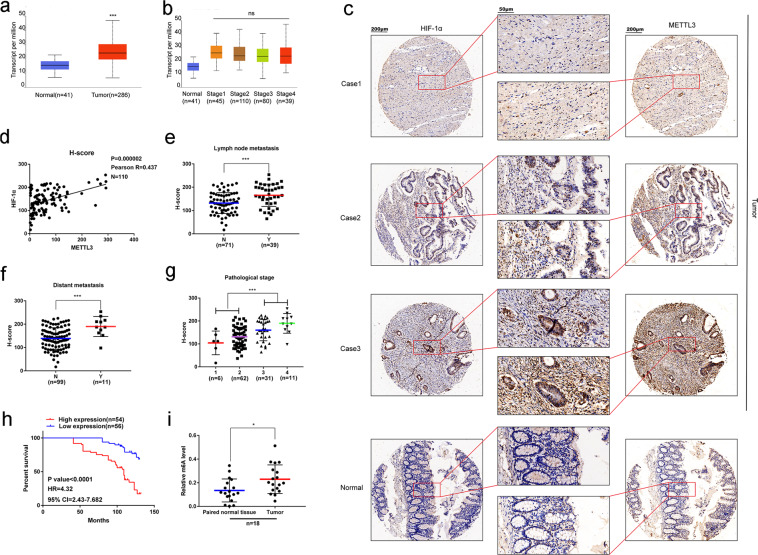
METTL3 and HIF-1α are co-expressed in colorectal cancer (CRC) accompanied with upregulated m6A modification. **a** The mRNA expression of METTL3 in TCGA-COAD databases. **b** Total m6A modification level in clinical CRC specimens and paired no-tumor bowel tissues (*n* = 18). **c** mRNA expression of METTL3 in each pathological stage of patients in TCGA-COAD databases. **d** Association of METTL3 and lymph node metastasis (N = no lymph node metastasis, *n* = 71; Y = lymph node metastasis, *n* = 39). **e** Association of METTL3 and distant metastasis (N = no distant metastasis, *n* = 99; Y = distant metastasis, *n* = 11). **f** Association of METTL3 and pathological stage (early stage: stage I and II, *n* = 68 vs. advanced stage: stages III and IV, *n* = 42). **g** Kaplan–Meier analysis showed high expression of METTL3 indicates poor overall survival (*p* < 0.0001, HR = 4.32, 95% CI = 2.43–7.682). (ns: no significance, *p < 0.05, ***p < 0.001). **h** Co-expression of METTL3 and HIF-1α in clinical CRC specimens analyzed by Pearson correlation analysis based on H-score (*P* = 0.000002, *R* = 0.437, *n* = 110). **i** The protein expression of METTL3 and HIF-1α in clinical CRC specimens and paired no-tumor bowel tissues performed by IHC (magnification: ×100 and ×400) (ns: no significance; *p < 0.05, ***p < 0.001)

Subsequently, we performed Western Blotting and RT-qPCR to determine the difference of m6A-associated enzymes between hypoxia and normoxia. The expression of METTL3, as well as the total m6A level, showed a significant increase in all four CRC cell lines with satisfying contingency in hypoxia (Supplementary Fig. S1a–c). Moreover, HIF-1α works as an important transcription factor in hypoxia that binds to HRE in the promoter of its target genes. By performing luciferase reporter gene assay and CHIP assay, we determined HIF-1α binds to two main HREs (−416 to 420; −35 to 38) in the promoter of METTL3, thus promotes its expression under hypoxia (Supplementary Fig. S1d–g). As HIF-1α, METTL3, METTL14 are all located at nuclear, and previous research reveals WTAP, a mammalian splicing factor, can interact with METTL3–METTL14 complex and affect this methylation. We wondered whether HIF-1α plays a similar role as WTAP, whereas a negative result was acquired (Supplementary Fig. S1h).

As HIF-1α is crucially responsible for Warburg effect, we further examined the expression of Warburg effect associated genes, ATP content, lactate production, glucose uptake, ECAR, and OCR under both normoxia and hypoxia. As expected, knockdown of METTL3 significantly repressed Warburg effect under both normoxia and hypoxia (Supplementary Fig. S2a–g). Remarkably, mRNA expression level of GAPDH, LDHA, Eno1, and HK2; protein expression level of GAPDH under normoxia showed a negative result. We inferred HIF-1α works as the key role in the repression of Warburg effect, and its rapid degradation under normoxia makes these negative results. Interestingly, knockdown of METTL3 repressed protein expression but not mRNA expression of METTL3. It seems METTL3 regulated the protein translation efficiency of HIF-1α.

MeRIP-qPCR was performed to investigate whether METTL3 regulated Warburg effect via m6A modification, and most Warburg effect associated genes showed abundant enrichment of m6A modification. A key m6A modification site at the CDS region (Chr14: 61738296) of HIF-1α was identified (Supplementary Fig. S3a–c). Subsequently, we constructed the HIF-1α CDS and HIF-1α CDS mutant (Chr14: 61738296A to C) to the luciferase reporter gene. We found that the translation efficiency of HIF-1α showed a significant decrease after knockdown of METTL3 both under hypoxia and normoxia (Supplementary Fig. S3d). YTHDF1 was reported as an important m6A reader responsible for the regulation of mRNA translation^2^. Thus, we performed RIP assay to determine whether YTHDF1 binds to HIF-1α. Significant enrichment of YTHDF1 was found in mRNA of HIF-1α, and hypoxia increased the enrichment level. Furthermore, overexpression of YTHDF1 rescued the inhibition of HIF-1α (Supplementary Fig. S3e). In addition, we detected the increased part of glucose uptake, lactate production, and ATP content after transfected with HIF-1α CDS and HIF-1α CDS mutant (Chr14: 61738296A to C) vectors in HCT-scr and HCT-shMETTL3. The difference of increasing part between HCT-scr and HCT-shMETTL3 showed significant depletion in mutant group compared with wide type group (Supplementary Fig. S3g–i). In summary, knockdown of METTL3 inhibited the translation efficiency of HIF-1α, thus repressed Warburg effect. We inferred similar inhibition of the translation efficiency also occurred in other Warburg effect genes. This explains why knockdown of METTL3 inhibited the protein expression of Glut1 and HK2, whereas showed unsatisfying inhibition to their mRNA expression.

Rapid energy intake is an important role of Warburg effect for the growth of tumors. Correspondingly, we demonstrated knockdown of METTL3 significantly repressed proliferation and clone formation in CRC cells (Supplementary Fig. S4a, b). In addition, we also demonstrated knockdown of METTL3 significantly inhibited the progression of CRC cells via Transwell assays and wound healing assay (Supplementary Fig. S4c, d). We inferred epithelial–mesenchymal transition (EMT), the classical progression mechanism, may contribute to this inhibition. On the one hand, knockdown of METTL3 inhibited HIF-1α which has been proved to be an important regulator for EMT. On the other hand, previous research has also demonstrated knockdown of METTL3 inhibited translation efficiency of Snail, an important EMT regulator via m6A modification^3^. In cell cycle analysis, we determined hypoxia-induced G0/G1 phase arrest. Furthermore, we also demonstrated that knockdown of METTL3 promoted the transition of G0/G1 phase to S phase, instead of promoting S phase to G0/M phase subsequently (Supplementary Fig. S4e). Combining with the result of proliferation inhibition, knockdown of METTL3 seems promoting the S phase arrest of CRC cells. Previous researches have demonstrated that depletion of HIF-1α causes an increased progression into S phase during hypoxia; however, there are also research found depletion of HIF-1α promotes G1 phase arrest during normoxia^4^. In addition, recent research has proved METTL3 participated in trans-lesion synthesis (TLS) to allow replication of past damaged lesions in S phase^5^. We suggested that METTL3 regulated cell cycle in CRC cells via m6A modification, in which process HIF-1α played an important role.

Finally, we demonstrated that knockdown of METTL3 inhibited the growth of xenografts in vivo accompanied with downregulated m6A modification level (Supplementary Fig. S5a, b). In addition, Warburg effect was an important basis for PET/CT examination. The uptake rate and concentration of contrast media (such as 18F-FDG) in tumors with active Warburg effect were higher. Through 18F-FDG PET, we visually demonstrated knockdown of METTL3 inhibited Warburg effect in vivo (Supplementary Fig. S5b–g).

In conclusion, our research demonstrated the regulation of m6A in both hypoxia and normoxia, as well as the interaction between HIF-1α, METTL3, and Warburg effect. We provided new insights for the metabolic mechanism of CRC based on m6A modification. The m6A writer METTL3 was determined as a potential diagnostic, prognostic marker, and potential therapeutic target.

## Supplementary information

Supplementary Material

## Data Availability

The datasets used and analyzed during the current study are available from the corresponding author on reasonable request.

## References

[1] Zhao BS, Roundtree IA, He C (2017). Post-transcriptional gene regulation by mRNA modifications. Nat. Rev. Mol. Cell Biol..

[2] Han D (2019). Anti-tumour immunity controlled through mRNA m6A methylation and YTHDF1 in dendritic cells. Nature.

[3] Lin X (2019). RNA m(6)A methylation regulates the epithelial mesenchymal transition of cancer cells and translation of Snail. Nat. Commun..

[4] Culver C, Melvin A, Mudie S, Rocha S (2011). HIF-1alpha depletion results in SP1-mediated cell cycle disruption and alters the cellular response to chemotherapeutic drugs. Cell Cycle.

[5] Xiang Y (2017). m6A RNA methylation regulates the UV-induced DNA damage response. Nature.

