# Supplementary material for: Knockdown of RNA N6-methyladenosine methyltransferase METTL3 represses Warburg effect in colorectal cancer via regulating HIF-1α
Source: Signal Transduct Target Ther. 2021 Feb 27;6:89. doi: 10.1038/s41392-021-00473-y (PMC7910535; doi:10.1038/s41392-021-00473-y)
Supplement: Supplementary file 1 — Supplementary Material [file 41392_2021_473_MOESM1_ESM.pdf]

Supplementary Materials for

Knockdown of RNA N6-methyladenosine methyltransferase METTL3 represses Warburg effect in colorectal cancer via regulating HIF-1α

Zhou Yang[1*], Yingjun Quan[2*], Yusheng Chen[1*], Yijun Huang[1], Renhong Huang[1], Weiping Yu[1], Dejun Wu[1], Min Ye[1#], Zhijun Min[1#], Bo Yu[1,3#]

[1]Department of General Surgery, Shanghai Pudong Hospital, Fudan University Pudong Medical Center, Shanghai 201399, China

[2]Department of General Surgery, Tongren Hospital, Shanghai Jiao Tong University School of Medicine, Shanghai 201399, China

[3]Department of General Surgery, Huashan Hospital Affiliated to Fudan University, Shanghai 201399, China

[*]Contributed equally to this work.

[#]Co-corresponding authors: Bo Yu, Zhijun Min and Min Ye, Department of General Surgery, Shanghai Pudong Hospital, Fudan University Pudong Medical Center, 2800 Gongwei Road, Huinan Town, Pudong, Shanghai, 201399, China; Email: yubo1217@126.com; minzhijun@126.com; yemincool@126.com.

This Supplementary file includes:

Materials and Methods
Figures S1 to S5
Tables S1 to S4

Material and methods

Patients and specimens

110 CRC specimens and paired non-tumor bowel tissues were collected from July 2017 to July 2019. Patients with the following criteria were excluded from participation: had received adjuvant chemotherapy or radiotherapy prior to surgery; had additional cancers diagnoses. All patients were classified according to the 7th edition of the TNM staging system 23. Postoperative adjuvant therapies were performed, according to standard schedules and doses. All participating patients gave their written informed consent. This study was approved by the Ethical Committee of Shanghai Pudong Hospital. The clinical data of all CRC patients was showed in Supplementary Table 2.

Immunohistochemical (IHC) staining

IHC was performed on paraffin-embedded sections. The sections were deparaffinized in xylene and hydrated with decreasing concentrations of ethanol (100, 90, 80, 75%) for 3 min each time and microwaved-heated in sodium citrate buffer for antigen retrieval. Then, the sections were blocked in 5% BSA and incubated with anti-METTL3 rabbit polyclonal antibody (1:100; Abclonal, Wuhan, China) and HIF-1α mouse monoclonal antibody (1:100; Novus Biologicals, USA) at 4°C overnight. Next, the sections were treated with horseradish peroxidase (HRP)-conjugated rabbit secondary antibody (1:200; ProteinTech Group, Inc., Wuhan, China) for 60 min at room temperature; then, 3,3’-diaminobenzidine development (DAB Substrate Chromogen System; Dako, Denmark) and hematoxylin staining were performed. The sections were fixed and images were obtained with inverted microscope (Olympus IX71, Japan).

An H-score was calculated using the following formula: H-score= $\sum (PI \times I)$ = (percentage of cells of weak intensity $\times 1$)+(percentage of cells of moderate intensity $\times 2$)+percentage of cells of strong intensity $\times 3$)[17]. Here H-score was recorded as a continuous variable.

RNA m6A quantification

Total RNA was extracted via TRIzol (Invitrogen, CA, USA) as described below, and RNA quality was assessed by NanoDrop (Thermo Fisher Scientific, Waltham, MA, USA). The m6A modification level of total RNA was examined via EpiQuik m6A RNA Methylation Quantification Kit (p-9005; Epigentek Group Inc., Farmingdale, NY, USA) according to the instruction. Briefly, 200 ng RNA accompanied with m6A standard were coated on assay wells, followed by capture antibody solution and detection antibody solution. The m6A levels were quantified colorimetrically by reading the absorbance of each well at a wavelength of 450 nm (OD450), and then calculations were performed based on the standard curve.

Cell Culture and hypoxia induce

Human CRC cell lines HCT116 and HT29, RKO, SW1116 were purchased from the University of Colorado Cancer Center Cell Bank. The cells were cultured in McCoy's 5A and RPMI 1640 medium respectively, supplemented with 10% FBS (Invitrogen, Carlsbad, CA, USA) at 37°C in a 5% CO_2 atmosphere. For hypoxia induce, cells were treated with cobalt chloride ($CoCl_2$) which prevents HIF-1α from degeneration by replacing the prolyl hydroxylase (PHD) cofactor Fe^{2+}[18]. In most analysis in this research, $CoCl_2$ was used at at 200μM for 24h, except for the following

analysis requiring prolonged hypoxia. In clone formation assay, $CoCl_2$ was used at at 100μM for 5 days; In cell proliferation assay, CoCl2 was used at at 100μM for 3 days. Additionally, in part assays, hypoxia was also induced by incubating in 1% O_2 for 24h in a three-gas incubator.

Knockdown and overexpression of METTL3 in CRC cell lines

The shRNA of human METTL3 (sequence: GCTGCACTTCAGACGAATTAT) was synthesized by Genomeditech,lnc. (Shanghai, China) and cloned into the pCDH-CMV-MCS-EF1-Puro lentiviral vector to construct the pCDH-shMETTL3 knockdown plasmid. In accordance with the instructions of the product manual, Lipofectamine 3000 (Invitrogen, Inc.) was used to co-transfect the target plasmid or the scrambled vector, psPAX2, PMG.2G into the HEK293T tool cells to obtain a METTL3 knockdown lentivirus or scrambled control lentivirus. Then, the lentivirus (multiplicity of infection, MOI=10) was used to infect HCT116 and HT29. The METTL3 knockdown cell lines HCT-shMETTL3/HT-shMETTL3 and negative control cell lines HCT-scr/HT-scr was screened by puromycin (2μg/mL, 72h). Similarly, construction of overexpressed METTL3 CRC cells and negative control were performing as described above via pCDH-CMV-MCS-EF1-Puro-METTL3 and pCDH-CMV-MCS-EF1-Puro empty plasmids respectively (named as HCT116-METTL3/HT29-METTL3 and HCT116-NC/HT29-NC). The knockdown and overexpression of METTL3 were confirmed by Western blotting.

Western Blotting Analysis

The total cellular proteins from each group were extracted using RIPA lysis buffer with 1% phenylmethanesulfonyl fluoride (PMSF). Then, equal amounts (20 μg) of protein determined by BCA protein assay kit (Thermo Fisher Scientific, Waltham, MA, USA) were separated using 10% SDS-PAGE gels. The proteins were then transferred to PVDF membranes (0.45 mm, Solarbio, Beijing, China). The membranes were blocked with 5% nonfat milk for 1 h at room temperature and then incubated with primary antibodies at 4°C for 12 h. The following antibodies were tested: METTL3, METTL14 (1:1000, Abclonal, Inc, China); WTAP, ALKBH5, FTO, YTHDF1, HIF-1α (1:1000, Proteintech Group. Inc); HK2, Glut1, GAPDH (1:4000, Abcam, UK) rabbit polyclonal antibodies. β-actin or α-tubulin rabbit polyclonal antibody (1:4000, Proteintech Group. Inc) was used as loading controls and normalization. The secondary antibodies were anti-mouse or anti-rabbit antibody and conjugated to horseradish peroxidase (HRP) (1:4000, Proteintech Group. Inc). The secondary antibodies were used at a 1:4000 dilution and were incubated for approximately 1 h at room temperature. The bands were visualized with ECL reagents (Thermo Fisher Scientific) and developed by Omega Lum G (Aplegen, USA).

RNA extraction, reverse transcription and quantitative PCR (RT-qPCR)

Total RNA was extracted by Trizol Regent (Invitrogen) from CRC cells. cDNA was obtained from total RNA with PrimeScript™ RT reagent kit (Takara Bio, Inc., Otsu, Japan). The mRNA expression was assessed by Real-time quantitative PCR, which was carried out in triplicate by a SYBR Premix Ex Taq™ kit (Takara Bio) and ABI 7900HT Real-Time PCR system (Applied Biosystems Life Technologies, Foster

City, CA, USA). The primers used are shown in Supplementary Table 3. The comparative cycle threshold values (2-ΔΔCt) were adopted to analyze the final results.

Luciferase reporter assay

The promoter and CDS of METTL3, as well as their mutant sequences were constructed into luciferase reporter vector pGL3-Rluc and followed by Dual-Glo Luciferase Assay system ((Promega Corp., Madison, WI, USA). After 36h transfection, the cells were lysed by passive lysis buffer. Firefly Luciferase (F-luc) and Renilla Luciferase (R-luc) of lysis were detected respectively. Transcription activity was represented as F-luc/R-luc, and translation efficiency was further normalized by mRNA expression of F-luc.

Co-Immunoprecipitation (Co-IP)

1 x 10^7 cells were harvested and lysed using NP-40 buffer. Lysates were pre-cleared by 20μL Protein A/G sepharose beads (Santa Cruz Biotechnology, USA) and centrifuged for supernatant. The pre-cleared lysate was added with 1μg METTL3, METTL14 (Abclonal), HIF-1α (ProteinTech Group. Inc) or IgG rabbit polyclonal antibody and incubated for 12 h at 4°C while rotating. Further 50 μl Protein A/G sepharose bead was added in lysate to capture the immunocomplex. After incubating for 4h at 4°C, the beads were harvested by centrifugation at 3000g for 3min, and washed four times with NP-40 buffer. Elution of the proteins was conducted by adding 2 x SDS loading buffer to the beads and boiling the for 5 min at 95°C. Subsequent, western blot was performed as described above.

Glucose uptake, lactate production and ATP content assays

For glucose uptake and lactate production assays, 5×10^5 cells were seeded onto 6-well plates and cultured at 37°C. After 24 h, 5 μl cell culture supernatant was collected in 96- well plates and mixed with 200 μl Glucose (HK) Assay Reagent or 100 μl Lactate Assay Regent (Sigma-Aldrich, CA, USA). After 20 minutes incubation at 37℃, the absorbance of 340 nm (OD_{340}) was determined with microplate reader. At the same time, standard curve is established through glucose and lactate standard. OD_{340} was substituted into standard curve to get glucose or lactate level. For ATP content assay, 2×10^3 cells were seeded onto 96-well plates and incubated for 48h. Culture medium was removed and ATPLite luminescent assay (PerkinElmer, Inc., Waltham, MA, USA) was added. After 30 minutes incubation at 37℃, luminescence was monitored. At the same time, standard curve is established through ATP standard. Luminescence intensity was substituted into standard curve to get ATP level. Glucose uptake, lactate production and ATP content were all normalized by protein concentration.

For the detection of lactate production and ATP content of subcutaneous xenografts, tissues were completely lysed by RIPA lysis buffer, followed by centrifuge for 14000*g*, 5min. Subsequently, the supernatant was harvested and performed assays as described above.

Extracellular Acidification Rate and Oxygen Consumption Rate Assays

The extracellular acidification rate (ECAR) and cellular oxygen consumption rate (OCR) of cells were measured using the Seahorse XFe 96 Extracellular Flux Analyzer (Seahorse Bioscience) via Glycolysis Stress Test Kit and Cell Mito Stress Test Kit respectively (Agilent Technologies, Santa Clara, CA, USA). Both assays were

performed according to the manufacturer's instructions. Briefly, 10^4 cells were seeded per well into a Seahorse XF 96 cell culture microplate for 24h, accompanied with or without treatment of $CoCl_2$. For ECAR, glucose (10mM), the oxidative phosphorylation inhibitor oligomycin (1μM), and the glycolytic inhibitor 2-DG (50mM) were sequentially injected into each well at the indicated time points; and for OCR, oligomycin(1μM), the reversible inhibitor of oxidative phosphorylation FCCP (p-trifluoromethoxy carbonyl cyanide phenylhydrazone, 1μM), and the mitochondrial complex I inhibitor rotenone plus the mitochondrial complex III inhibitor antimycin A (Rote/AA, 1μM) were sequentially injected. All data was normalized by protein concentration via BCA assay as described above.

MeRIP-qPCR

Intact total RNA was extracted via centrifugation column (MiniBEST Universal RNA Extraction Kit; Takara) and mRNA was further purified via polyATtract mRNA Isolation Systems (Promega Corp.). Subsequently, m6A RNA immunoprecipitation (MeRIP) was performed with Magna MeRIP m6A kit (17-10499, Millipore) according to the manufacturer's instructions. The IP production was performed RT-qPCR as described above. The primers for RT-qPCR were showed in Supplementary Table 4.

RNA immunoprecipitation (RIP)

For RIP assay, cells were irradiated twice with 400 mJ/cm^2 at 254 nm by Stratalinker on ice and lysed with RIP lysis buffer (300 mM NaCl, 0.2% NP-40, 20 mM Tris-HCl PH 7.6, 0.5 mM DTT, protease inhibitor and RNase inhibitor) at 4 °C through disruptive sonication. Then the lysis was incubated with 5μg anti-YTHDF1 Rabbit

antibody, or IgG (ProteinTech Group) pre-conjugated protein A/G Magnetic Beads (Millipore) in 500 μl IP buffer (150 mM NaCl, 10 mM Tris–HCl (pH 7.4), 1 mM EDTA, 1 mM EGTA, 1 % Triton X-100, 0.5 % NP-40) supplemented with RNase inhibitors (Thermo Fisher) at 4 °C overnight. The IP complex was treated with Proteinase K (Thermo Fisher) for 1h at 52 °C, and RNA was purified with phenol:chloroform:isoamyl alcohol. Finally, RT-qPCR was performed as described above.

Clone formation test

For plate clone formation assay, 500 cells were seeded into 6-well plates and incubated at 37°C. Clone size was observed daily under a microscope until the number of cells in the majority of clones was >50. Then, the medium was removed and the cells were stained with 0.2% crystal violet for 30 min. The cells were washed 3 times with PBS, then photographed and the clones were counted. The ratio of clone formation was calculated with the following equation: Ratio of clone formation (%) = clone number / 500 x 100.

For clone formation assay applicated with limited dilution, cells were continuously diluted to 1 cell per 100ul RPMI-1640 and seeded in 96-well plate. Clone size was observed daily under a microscope until the number of cells in the majority of clones was >50. Image was captured and cell number was counted directly in each clone.

Cell proliferation assay

3×10^3 cells suspended in 100ul RPMI-1640 medium were seed into 96-well plate. The cell proliferation was assessed by the CCK8 (Dojindo Molecular Technologies,

Japan). 10ul CCK8 solution was given to each well of the plate after different incubation times: 0h, 24h, 48h and 72h. Finally, we measured the absorbance at 450nm wavelength after 2h incubation.

Cell migration and invasion assays

Cell migration and invasion were analyzed with transwell plates (24-well insert, 8 μm pore size; BD Biosciences, Bedford, MA, USA). The filters (Corning Inc., USA) were coated with (invasion assay) or without (migration assay) 55 μL Matrigel (1:8 dilution; BD Biosciences). The 10^4 cells were suspended in 100μl RPMI-1640 medium without serum and seeded in the upper chamber. Next, 600μl 90% RPMI-1640 supplement with 10% FBS was added to the bottom chamber. After incubation for 24h, the chambers were fixed by 4% paraformaldehyde for 30 min and then stained by 0.1% crystal violet for 30 minutes. At last, we used a magnification microscope to count the amount of the invasion cells in the bottom of the chamber.

Wound healing assay

5×10^5 cells were seeded into 6-well culture plates and sterile micro-pipette tip was used to scratch the confluent monolayers in the shape of a straight line. The cells were incubated to cover the wound for 24 h and pictures of the same wound was taken under a microscope. The wound areas were analyzed by Image J software.

Cell Cycle assay

For cell cycle assay, $1x10^6$ cells were harvested, fixed in 70% ethanol, and stored at 4˚C overnight. Cells were then stained with PI staining solution for 30 min in the

dark at room temperature followed by flow cytometry. The fractions of the cells in G1, S, and G2/phases were calculated with Modfit software (Verity Software House, USA).

Subcutaneous xenografts of nude mice

5-week-old male Balb/c-nu mice were provided by the Beijing Vital River Laboratory Animal Technology Co. Ltd. All detailed experimental procedures were approved by the Institutional Animal Care and Utilization Committee of Fudan University Pudong Animal Experimental Center. All the mice (n = 12) were equally and randomly divided into the HCT-scr and HCT-shMETTL3 group. 3×10^6 HCT-scr or HCT-shMETTL3 cells suspended in 100μl PBS were injected subcutaneously from the axilla of each nude mice. After 1 weeks, the long (L) and short (S) diameter of the tumors were measured with vernier caliper every 3 days (tumor volume=$L*S^2/2$). The growth curve of subcutaneous tumors was drawn on the basis of the measured tumor volume. All mice were killed after 3 weeks since injection of CRC cells and subcutaneous tumors were removed completely. The tumors were weighed and performed into paraffin section.

PET imaging of glucose uptake in mice

PET imaging of mice was performed using an animal PET scanner (Siemens Corp.) before the sacrifice at the third week. Mice were injected intravenously with 3.7 MBq (100 mCi) of 18F radio-labeled fluorodeoxyglucose (18F-FDG) after anesthetization with pentobarbital. Five-minute emission scans were performed to obtain attenuation correction data in the prone position at 60 minutes after injection, and delay scans of 10 minutes were acquired at 2 hours.

Chromatin immunoprecipitation (CHIP) assay

CHIP was performed according to the manufacturers' instructions by using Magna CHIP Kit (Millipore, Darmstadt, Germany). Chromatin samples were immunoprecipitated with antibodies against a negative control normal Rabbit IgG, HIF-1α (2μg, Novus Biologicals). Subsequently, IP production was performed with RT-qPCR as described above. The primers of METTL3 for CHIP analysis were as follows: Forward: 5'-GTGATTCCCGAAATGTTT-3' and reverse: 5'-TCCTGGATTCTAGCTGCC-3'.

Statistical analysis

All the experiments were performed 3 times at least. SPSS software (version 19.0, IBM Corp., Armonk, NY, USA) was used for statistical analysis of all the experimental data. GraphPad Prism (version 7, GraphPad Software, La Jolla, CA, USA) was used to determine the statistical results. All data are expressed as the mean + standard deviation (mean + sd). The statistical analysis of the data from 2 groups was performed using a t-test. The comparisons of multiple groups were performed by one-way ANOVA and then an LSD-t test. $P < 0.05$ was considered to be significant.

Figure S1

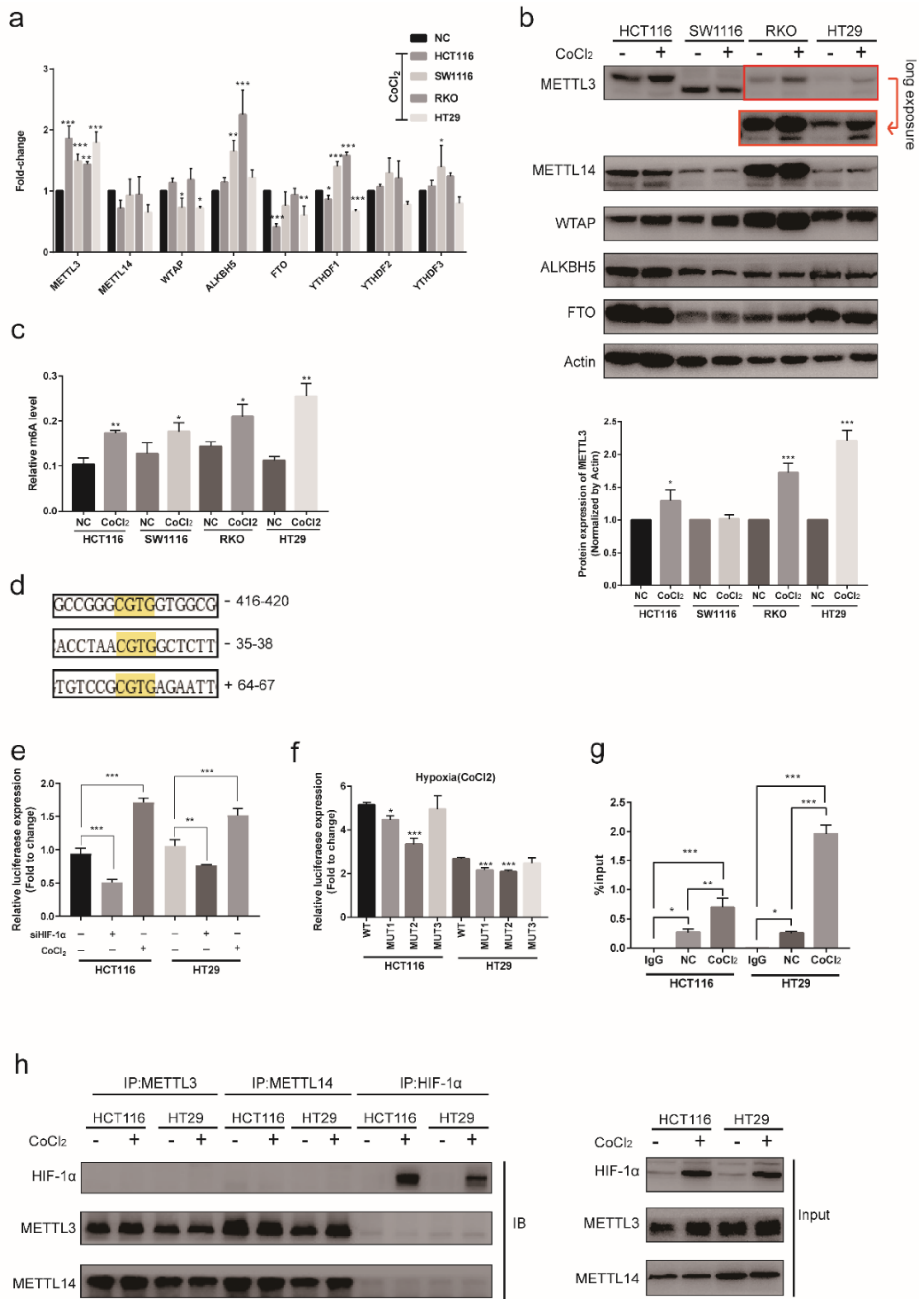

Figure S1. HIF-1α binds to the promoter of METTL3 and increased its expression at transcriptional level. A. The mRNA expression of m6A associated enzymes (METTL3, METTL14, WTAP, ALKBH5, FTO, YTHDF1/2/3) in CRC cell lines (HCT116, SW1116, RKO, HT29) under both normoxia and hypoxia performed by RT-qPCR. NC groups of each cell line were simplified to "NC". B. The protein expression of m6A associated enzymes (METTL3, METTL14, WTAP, ALKBH5, FTO) in CRC cell lines under both normoxia and hypoxia performed by Western Blotting. C. Total m6A modification level of CRC cell lines under both normoxia and hypoxia. D. Possible HREs (-CGTG-) in the promoter of METTL3. E. Luciferase expression of METTL3 promoter in HCT116 and HT29 treated with $CoCl_2$ and siHIF-1α. F. Luciferase expression of different mutant HREs (MUT1: -416-420, MUT2: -35-38, MUT3: +64-67) in METTL3 promoter under hypoxia. G. Transcriptional activity of HIF-1α at promoter of METTL3 performed by CHIP analysis. H. Interaction of HIF-1α with METTL3/METTL14 complex performed by Co-IP. (*p<0.05, **p<0.01, ***p<0.001).

Figure S2

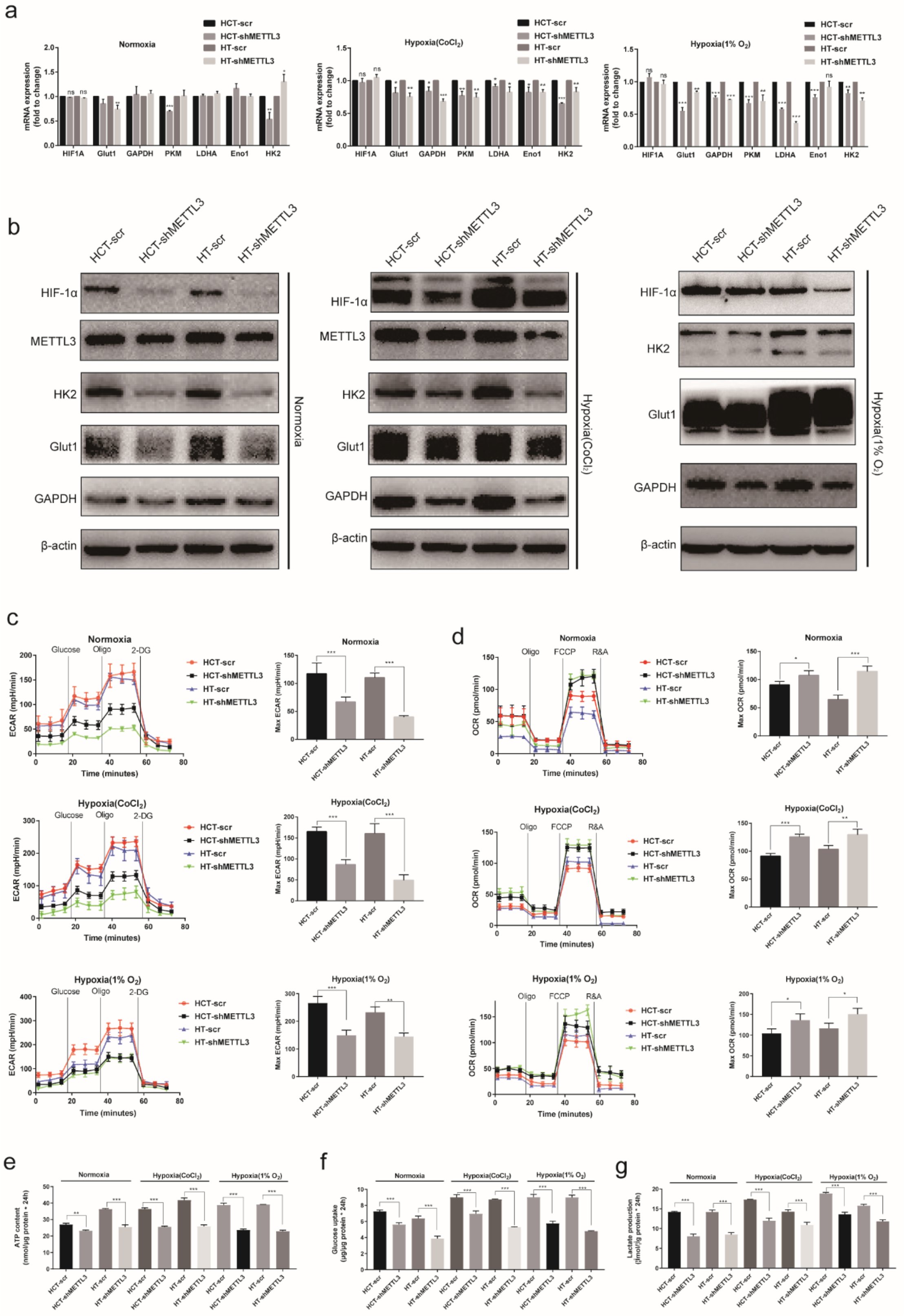

Figure S2. Knockdown of METTL3 inhibits Warburg effect in CRC both under hypoxia and normoxia. A. The mRNA expression of Warburg effect associated genes (HIF1A, Glut1, GAPDH, PKM, LDHA, Eno1, HK2) in CRC cells (HCT-scr/shMETTL3, HT-scr/shMETTL3) under normoxia and hypoxia (induced by $CoCl_2$ or 1% O_2). B. The protein expression of Warburg effect associated genes (HIF-1α, HK2, Glut1, GAPDH) of CRC cells under both

normoxia and hypoxia. C. Extracellular acidification rate (ECAR) of CRC cells under both normoxia and hypoxia performed by Searhorse XF96 analysis. D. Cellular oxygen consumption rate (OCR) of CRC cells under both normoxia and hypoxia performed by Seahorse XF96 analysis. E. ATP content of CRC cells. F. Glucose uptake of CRC cells. G. Lactate production of CRC cells. (ns: no significance. *p<0.05, **p<0.01, ***p<0.001)

Figure S3

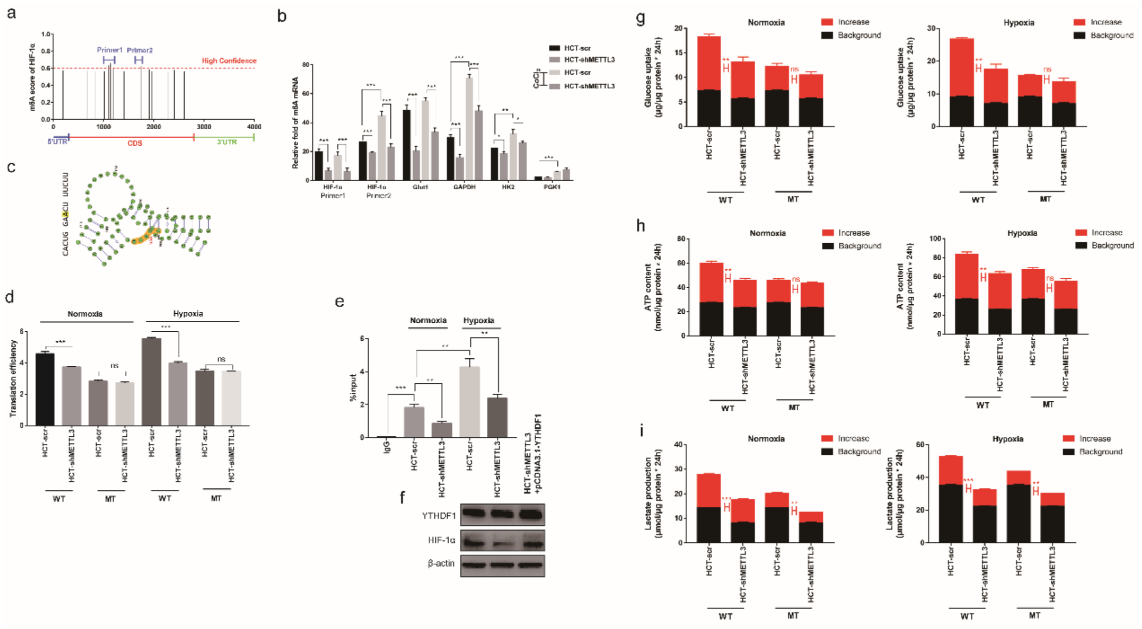

Figure S3. Knockdown of METTL3 repressed translation efficiency of HIF-1α via m6A modification. A. m6A modification site of HIF-1α predicted by SRAMP website tools based on sequence-derived features (High Confidence: score > 0.6), and primers designed for MeRIP-qPCR. B. m6A modification of Warburg effect genes in HCT-scr and HCT-shMETTL3 under both normoxia and hypoxia performed by MeRIP-qPCR. C. Most abundant enrichment of m6A modification cite of HIF-1α: Chr14: 61738296. D. Translation efficiency of HIF-1α in HCT-scr and HCT-shMETTL3 under both normoxia and hypoxia performed by luciferase reporter gene (WT: CDS region of HIF-1α, MT: Chr14: 61738296 mutant (A-C) CDS of HIF-1α). E. Enrichment of HIF-1α mRNA in YTHDF1 protein both under normoxia and hypoxia performed by RIP assay. F. Expression of YTHDF1 in HCT-scr and HTC-shMETTL3 under hypoxia and its rescue effect to HIF-1α. G. Glucose uptake of CRC cells (HCT-scr, HCT-shMETTL3) transfected with wide type (WT)/mutant type (MT) HIF-1α CDS under both normoxia and hypoxia. H. ATP content of CRC cells transfected with wide type (WT)/mutant type (MT) HIF-1α CDS under both normoxia and hypoxia. I. Lactate production of CRC cells transfected with wide type (WT)/mutant type (MT) HIF-1α CDS under both normoxia and hypoxia. (ns: no significance, *p<0.05, **p<0.01, ***p<0.001).

Figure S4

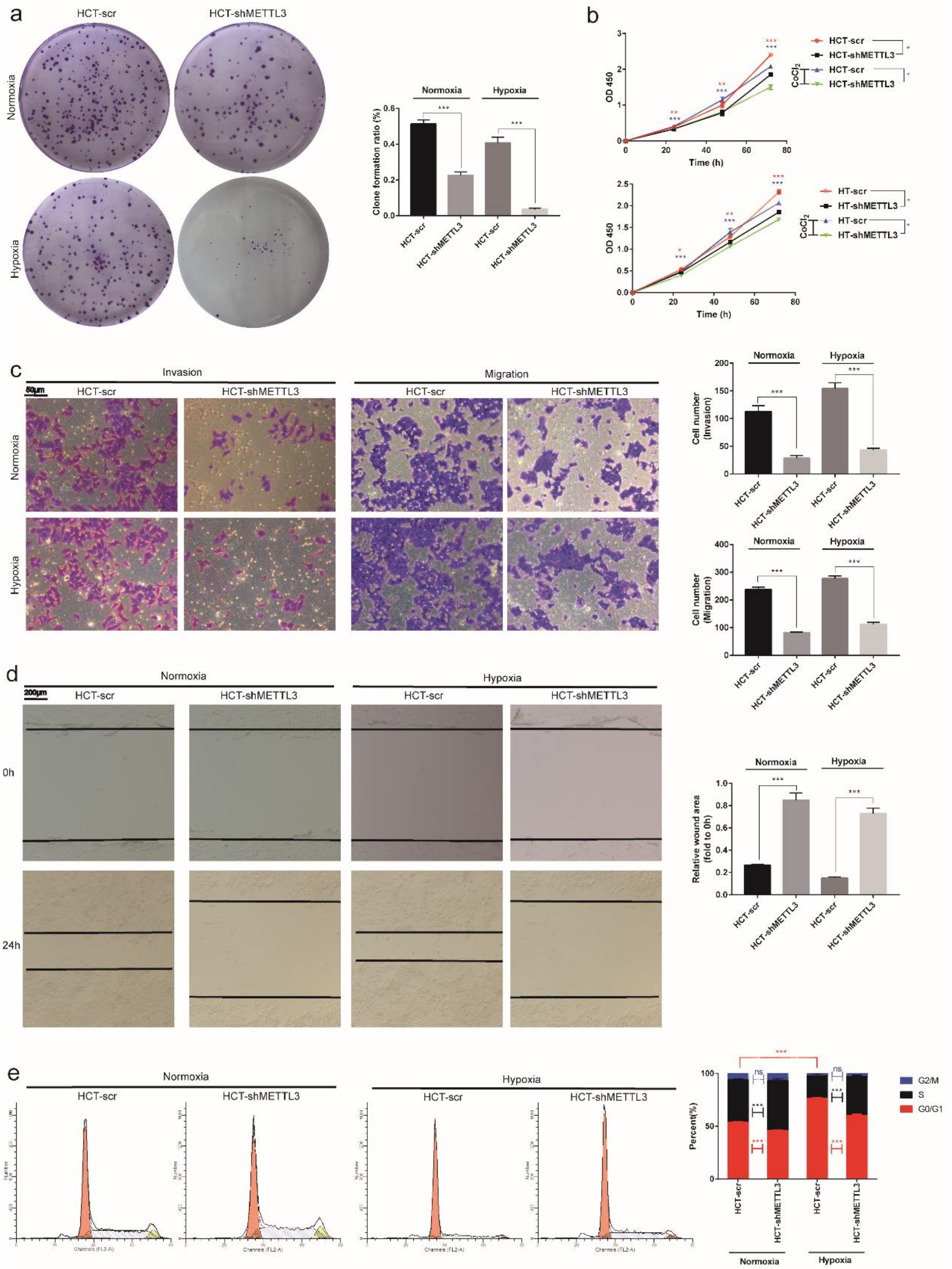

Figure S4. Knockdown of METTL3 repressed CRC growth, progression and percentage of G0/G1 phase in vitro. A. Clone formation ability of HCT-scr and HCT-shMETTL3 under both normoxia and hypoxia. B. Proliferation ability of HCT-scr and HCT-shMETTL3 under both normoxia and hypoxia performed by CCK8. C. Invasion and migration ability of HCT-scr and HCT-shMETTL3 under both normoxia and hypoxia performed by Transwell assays (magnification: 400x). D. Migration ability of HCT-scr and HCT-shMETTL3 under both normoxia and hypoxia

performed by wound healing assay (magnification: 100x). E. Cell cycle of HCT-scr and HCT-shMETTL3 under both normoxia and hypoxia performed by flowcytometry. (ns: no significance, *p<0.05, **p<0.01, ***p<0.001)

Figure S5

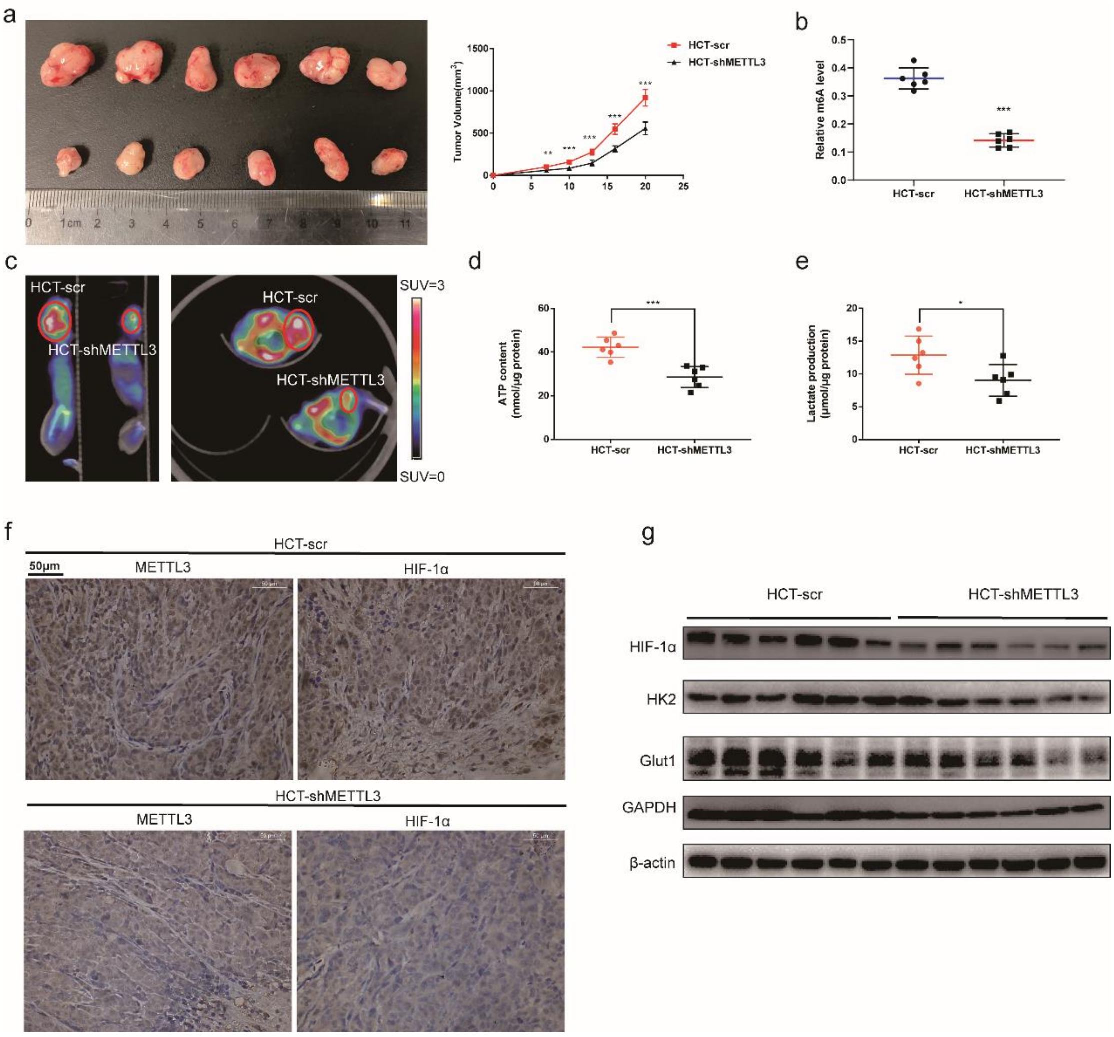

Figure S5. Knockdown of METTL3 repressed CRC growth and Warburg effect in vivo. A. Subcutaneous xenografts growth curve and tumor weight of each group (HCT-scr and HCT-shMETTL3 group). B. Total m6A modification level of removed xenografts. C. PET/CT image performed before sacrifice of mice in each group (SUV: standard uptake value). D. ATP content of removed xenografts in each group. E. Lactate production of removed xenografts in each group. F. Expression and location of METTL3 and HIF-1α of removed xenografts performed by IHC.(magnification: 400x). G. The protein expression of Warburg effect associated genes (HIF-1α, HK2, Glut1, GAPDH) in removed xenografts. (*p<0.05, **p<0.01, ***p<0.001).

Table S1 Cox's proportional hazard model of CRC patients

Factors	Univariate analysis			Multivariate analysis		
	P-value	HR	95% CI	P-value	HR	95% CI
Age	0.457	1.007	0.989-1.024			
Sex	0.201	0.907	0.907-1.595			
T	0.016	1.829	1.118-2.994			
N	0.000	5.117	3.375-7.760	0.000	3.965	1.953-8.049
M	0.000	6.096	2.886-12.968	0.041	2.298	1.034-5.109
Histological type	0.507	1.236	0.661-2.313			
METTL3	0.000	5.272	2.829-9.824	0.000	3.743	1.938-7.228
HIF-1a	0.521	0.837	0.487-1.439			

Table S2. Clinical characteristics of patients

Tumor(T) stage	pT1	0
	pT2	6
	pT3	50
	pT4	54
N stage	N0	71
	N1	26
	N2	13
M stage	M0	99
	M1	11
Age	≤60	62
	> 60	48
Gender	Male	74
	Female	36
Tumor location	Right colon	39
	Left colon	22
	Transverse colon	15
	Sigmoid colon	34
Histological grade	Well Differentiated	92
	Poorly Differentiated	18
Mucinous Colloid Type	No	88
	Yes	22

Table S3. The Primers of RT-qPCR

Gene	Forward Primer	Reverse Primer
**METTL3**	AAGCTGCACTTCAGACGAAT	GGAATCACCTCCGACACTC
**METTL14**	AGAAACTTGCAGGGCTTCCT	TCTTCTTCATATGGCAAATTTTCTT
**WTAP**	GGCGAAGTGTCGAATGCT	CCAACTGCTGGCGTGTCT
**ALKBH5**	CCCGAGGGCTTCGTCAACA	CGACACCCGAATAGGCTTGA
**FTO**	TGGGTTCATCCTACAACGG	CCTCTTCAGGGCCTTCAC
**YTHDF1**	ACCTGTCCAGCTATTACCCG	TGGTGAGGTATGGAATCGGAG
**YTHDF2**	GGCAGCACTGAAGTTGGG	CTATTGGAAGCCACGATGTTA
**YTHDF3**	TCAGAGTAACAGCTATCCACCA	GGTTGTCAGATATGGCATAGGCT
**Actin**	GGGACCTGACTGACTACCTC	TCATACTCCTGCTTGCTGAT
**PKM**	ACTGGCATCATCTGTACCATTG	AGCCACATTCATTCCAGACTTA
**LDHA**	GGTTGGTGCTGTTGGCATGG	TGCCCCAGCCGTGATAATGA
**HK2**	CGACAGCATCATTGTTAAGGAG	GCAGGAAAGACACATCACATTT
**Glut1**	CTGGCATCAACGCTGTCTTC	GCCTATGAGGTGCAGGGTC
**Eno1**	GCCTCCTGCTCAAAGTCAAC	AACGATGAGACACCATGACG
**PGK1**	TTCTGTTCTTGAAGGACTGTGT	CTTTAACCTTGTTCCCAGAAGC
**GAPDH**	GTCAAGGCTGAGAACGGGAA	AAATGAGCCCCAGCCTTCTC
**HIF-1α**	GAACGTCGAAAAGAAAAGTCTCG	CCTTATCAAGATGCGAACTCACA

Table S4. The Primers of MeRIP-qPCR

Gene	Forward Primer	Reverse Primer
**HIF-1α (848)**	TTTCCTCAGTCGACACAGCC	TCCACCTCTTTTGGCAAGCA
**HIF-1α (1458)**	ATGTCTCCATTACCCACCGC	ATCCTGAATCTGGGGCATGG
**HK2**	GTTGCTTCTGGCTCCTCCTT	ATTTTAGGACAGAGGCGGGC
**Glut1**	TGCCTGAGGGTGGAGACTAA	AAGGAGACTAGAACCCGGCA
**GAPDH**	GACCACAGTCCATGCCATCA	GTCAGGTCCACCACTGACAC
**PGK1**	CACTGTGGCTTCTGGCATAC	CACACAATCTGCTTAGCCCG
